# A Case Report on Fever of Unknown Origin in a 10-Year-Old: Tubercular Liver Abscess

**DOI:** 10.7759/cureus.56319

**Published:** 2024-03-17

**Authors:** Sumita Biswas, Md Wahiduzzaman Mazumder, Uma Gupta, Purna Talukder, MD. Omar Faruk

**Affiliations:** 1 Pediatrics, Bangabandhu Sheikh Mujib Medical University, Dhaka, BGD; 2 Pediatric Gastroenterology, Bangabandhu Sheikh Mujib Medical University, Dhaka, BGD; 3 Internal Medicine, Interfaith Medical Center, New York, USA

**Keywords:** fever of unkown, ascitic fluid analysis, : tuberculosis, : liver abscess, pediatric gastroenterology

## Abstract

The liver, which presents as a focal point for tuberculosis in pediatric cases, is rarely encountered, and reported instances are scarce. This atypical manifestation underscores the management of tuberculosis affecting this particular organ in the context of pediatric patients. The treatment of solitary tubercular liver abscesses in children necessitates a collaborative approach, engaging pediatricians, infectious disease specialists, and interventional radiologists. It also needs awareness among physicians to explore and treat early and to complete further assessments for a better outcome. In our instance, investigating the cause of fever led us to diagnose a tubercular liver abscess in a previously healthy 10-year-old male. The substantiation of this diagnosis was accomplished through a meticulous liver biopsy, wherein immunohistochemistry was employed to detect tubercular pathogens. Following the confirmation of the diagnosis, the initiation of a targeted therapeutic regimen resulted in the subsequent resolution of the fever.

## Introduction

Fever of unknown origin (FUO) in children is defined as a fever exceeding 38.3°C (101°F) occurring at least once a day for at least eight days without any clear diagnosis following the initial outpatient or hospital evaluation, which includes a comprehensive history, thorough physical examination, and initial lab tests [1]. Liver abscess is an uncommon disease in children; it is very rare in developed countries but occurs frequently in children in developing countries [2]. Tubercular liver abscess is usually associated with immune-compromised patients, and it is not so frequent without active pulmonary or military tuberculosis or any focus in the gastrointestinal tract [3]. Due to the rarity of tubercular liver abscesses in children and the diagnostic challenges associated with them, only a few cases have been reported in the literature. Most of the cases are documented in adult patients. This case report presents the clinical findings and management of a 10-year-old male who was admitted with a history of unexplained fever, weight loss, and abdominal distension. The purpose of this report is to highlight the challenges faced in diagnosing and managing such a case and to emphasize the importance of a thorough evaluation to reach an accurate diagnosis.

## Case presentation

This case report presents the clinical findings and management of a 10-year-old male who was admitted with a one and half a month history of unexplained fever, weight loss, and abdominal distension. The fever initially started as a low-grade fever but progressed to high-grade and intermittent episodes, more pronounced in the evenings. He experienced a documented, significant 10% weight loss during the six-week duration. He also complained of right-sided upper abdominal pain, which was dull and diffuse in nature.

**Figure 1:**
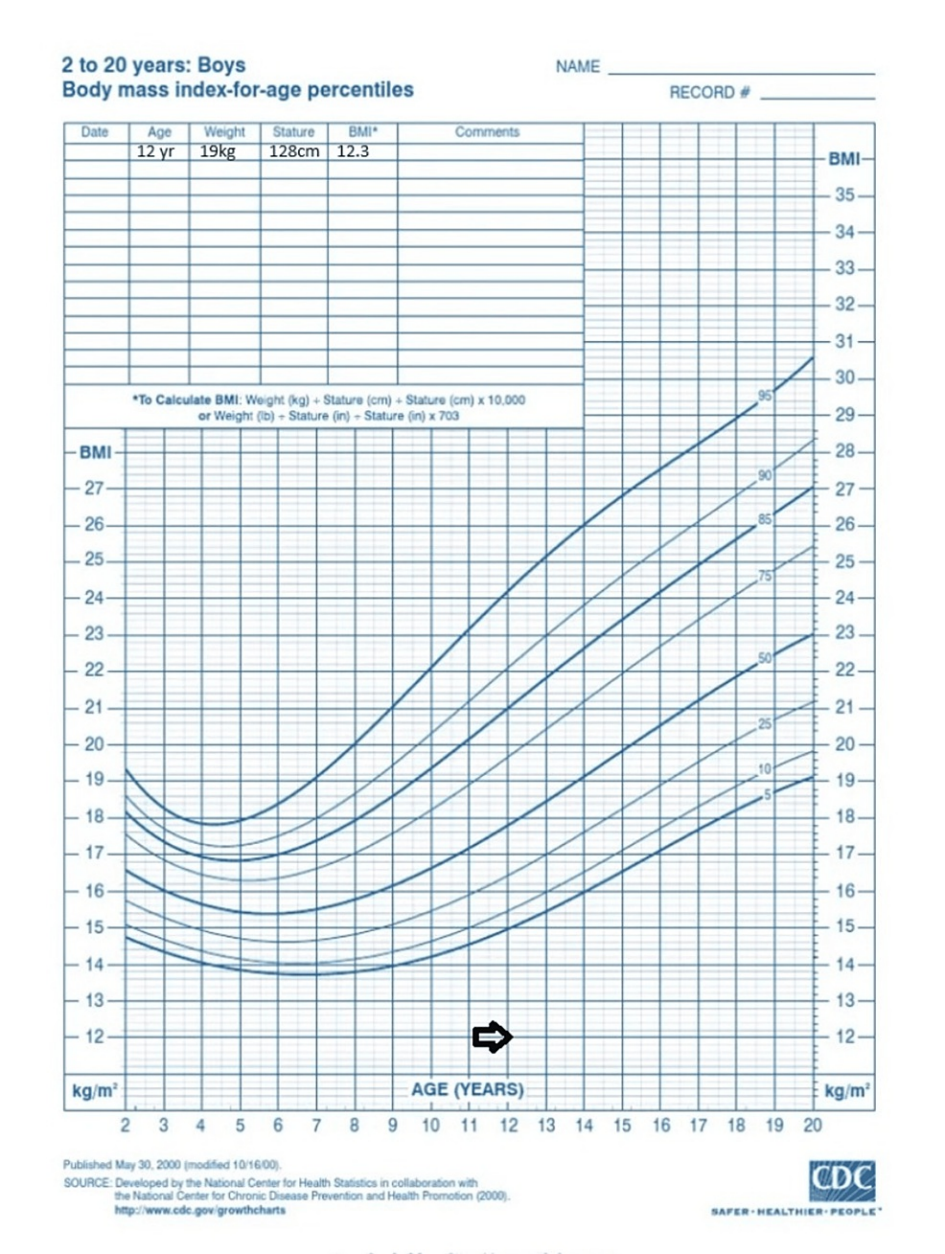
The patient's weight was 19 kg, his height was 128 cm, and his BMI was 12.3 kg/m2, which falls below the third percentile according to CDC guidelines Kg = Kilogram; cm= Centimeter; Kg/m^2^=Kilogram/meter^2^

He had no history of jaundice, cough, breathing difficulty, or close contact with tuberculosis (TB) patients. There were no signs of progressive pallor, night sweats, palpable lymphadenopathy, body ache, headache, or bleeding manifestations. A physical examination revealed a toxic and sick-looking appearance with mild pallor. The patient was anthropometrically severely underweight (Figure 1). There were no signs of jaundice, cyanosis, clubbing, koilonychia, dehydration, or edema. The oral cavity appeared healthy. Abdominal examination showed a distended abdomen with a centrally placed and everted umbilicus. Flanks appeared full with moderate ascites-no organomegaly-and visible peristalsis and engorged veins were absent.

Further investigations were conducted, including blood tests, imaging studies, and ascitic fluid analysis. The laboratory findings showed mild normochromic normocytic anemia, no leukocytosis (lymphocytosis: 3200/cumm), elevated alkaline phosphatase, normal liver enzymes (albumin 3.1 g/L, bilirubin 0.6 mg/dl), and an elevated erythrocyte sedimentation rate (ESR) (Table 1).

**Table 1:** Significant lab values MTB: Mycobacterium tuberculosis

Parameters	Results	Normal Range
Hemoglobin (Hb)	9.8 g/dl	11.5-15.5 gm/dl
Erythrocyte Sedimentation Rate (ESR)	76 mm	0—10 mm
White blood cell (Wbc)	7,000/cumm	4,000-10,000/cumm
Differentials	Neutrophil (N) :41%, Leukocyte (L) : 58%, Monocyte:1%	N: 45-55% L: 20-30%
Alkaline Phosphatase (ALP)	383	Upto 340 U/L
Prothrombin time/(PT/INR)	13.5/1.21	
Gamma Glutamyl Transpeptidase (GGT)	83 U/L	12-58 U/L
Peripheral Blood Film (PBF)	Microcytic Hypochromic Anemia	
Lactate Dehydrogenase (LDH)	332 U/L	120-246 U/L
Serum Glutamic Pyruvate Transaminase (SGPT)	39 U/L	10-49 U/L
C- Reactive protein (CRP)	46.81 mg/L	<5 mg/L
Serum Creatinine	0.32 mg/dl	0.6-1.2 mg/dl
Sputum for Gene X-pert	MTB not detected	
Blood Culture	Negative	

An ascitic tap was conducted with the aim of ruling out malignancy and liver cirrhosis; however, the low serum-ascites albumin gradient (SAAG < 1.1 g/dL) proved the absence of portal hypertension and pointed towards a peritoneal source for the accumulation of ascitic fluid. It was inconclusive, as no *Mycobacterium tuberculosis* (MTB) was detected, though leukocytosis was observed. This finding suggests several potential conditions affecting the peritoneum, including primary peritoneal mesothelioma, secondary peritoneal carcinomatosis, tuberculous peritonitis, and infections caused by fungi or parasites. Additionally, the patient exhibited positive findings for questionable malignant cells, warranting further investigation through a liver biopsy (Table 2).

**Table 2:** Summary of positive findings in ascitic fluid

Parameters	Results	Normal
Physical Examination: Colour Appearance	Straw Hazy	Straw Clear
Microscopic Examination Total cell count Neutrophils Lymphocytes	800 cells/cumm 15% 85%	50-200 c/cumm 0-1% 0-1%
Gram Stain	Some Gram-positive cocci	Negative
Ziehl-Neelson stain	Not found	Negative
Biochemistry: ADA (Adenosine deaminase) Albumin Glucose Protein	20.48 U/L 12.32 g/L 5.2 mmol/L 31 g/dl	0-24 U/L 2-8 g/L 7-10mmol/L 0.3-4 g/dl
Histopathology	Negative for malignancy	Negative
Cyto-spin findings of ascitic fluid	Positive for malignancy	Negative
Gene X-pert test	MTB not found	Non reactive

Ultrasound of the abdomen revealed that in the liver, the organ is mildly enlarged in size (12 cm) and regular in outline. Hepatic parenchyma is homogenous in echotexture with uniformly fine-level echoes. Multiple loculated cystic lesions are noted in both lobes of the liver, measuring about 70mm × 28 mm, 20mm × 16 mm, 17mm × 17 mm, and 36 mm × 27 mm. The portal vein, hepatic vein, and their tributaries are normal. Porta hepatis appears normal with moderate ascites. However, serial ultrasound was done, and the findings gradually improved after the initiation of appropriate treatment.

Chest X-ray with normal findings throughout the treatment cycle.

**Figure 2:**
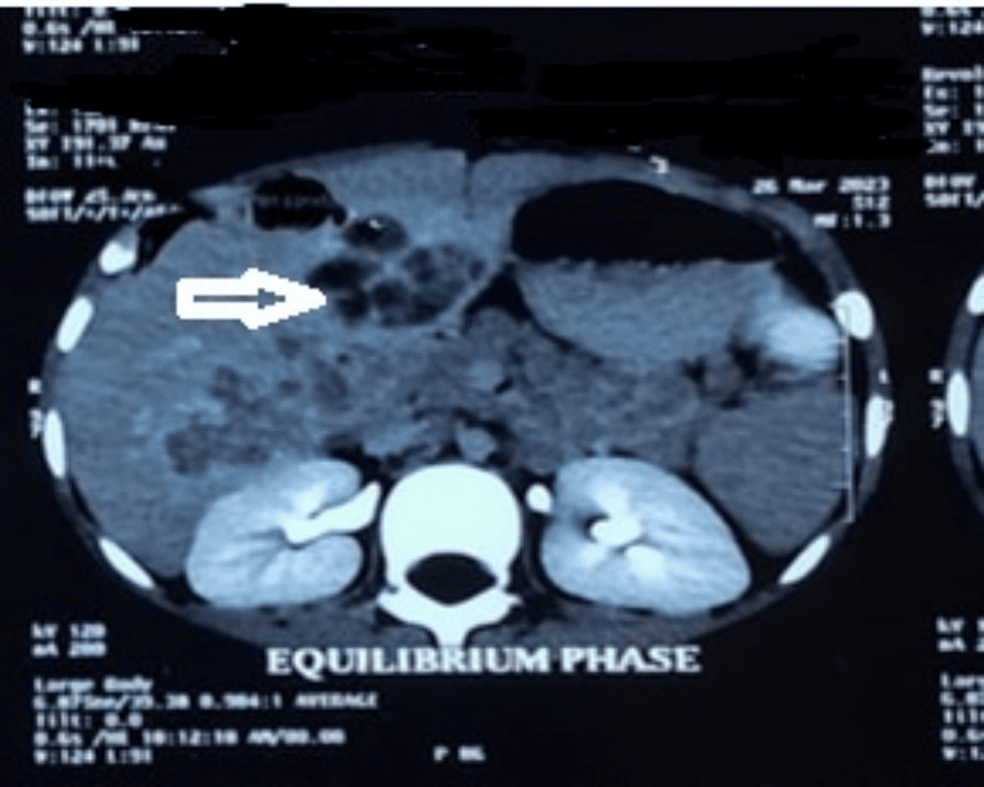
A CT scan confirmed the presence of multiple cystic hepatic lesions with multiloculation and some air density. Marginal enhancement with septations, septal enhancement, and mildly reactive adjacent parenchymal tissue

**Figure 3:**
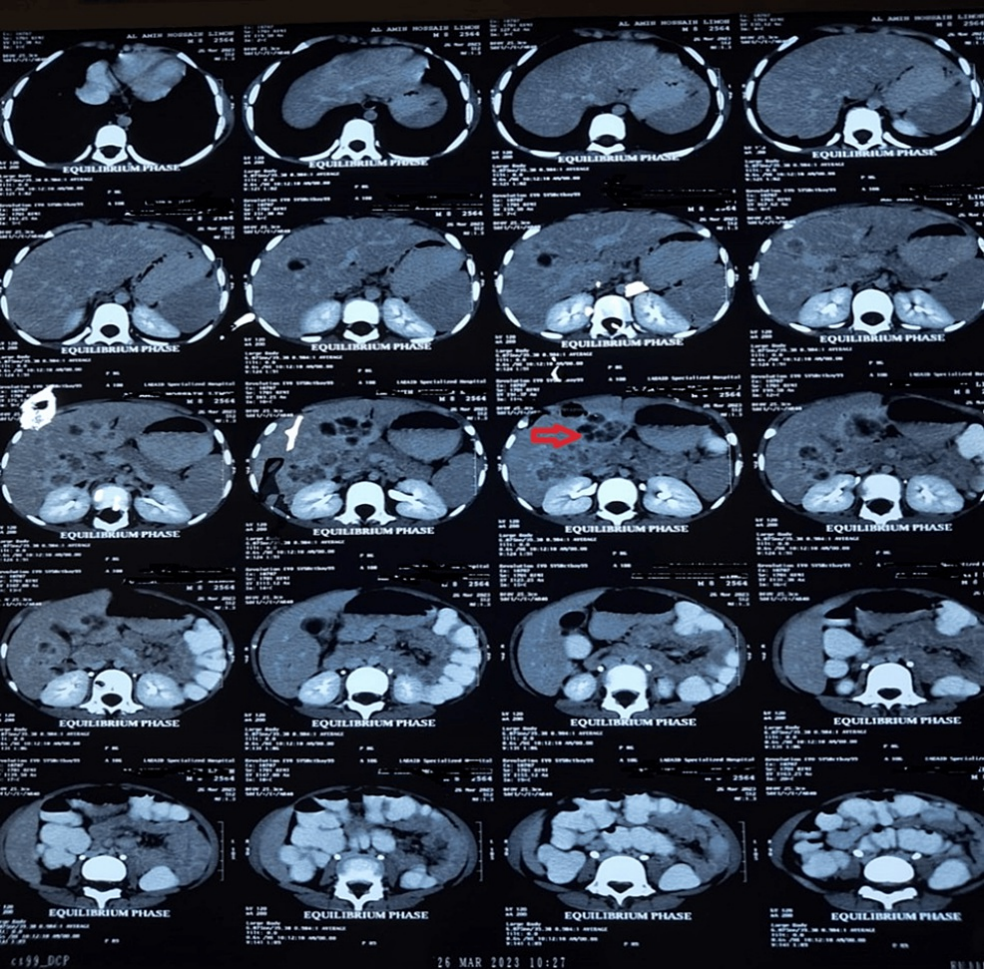
Contrast CT scan of the hepatobiliary system

CT findings of the hepatobiliary system (HBS) are suggestive of hepatomegaly with fatty change and multiple cystic hepatic space-occupying lesions (SOLs) with multiloculations and some air density involving both lobes. There is no portovenous thrombosis. Mild splenomegaly. Trace amount ascites. Small porta-hepatic lymph nodes (Figures 2, 3).

**Figure 4:**
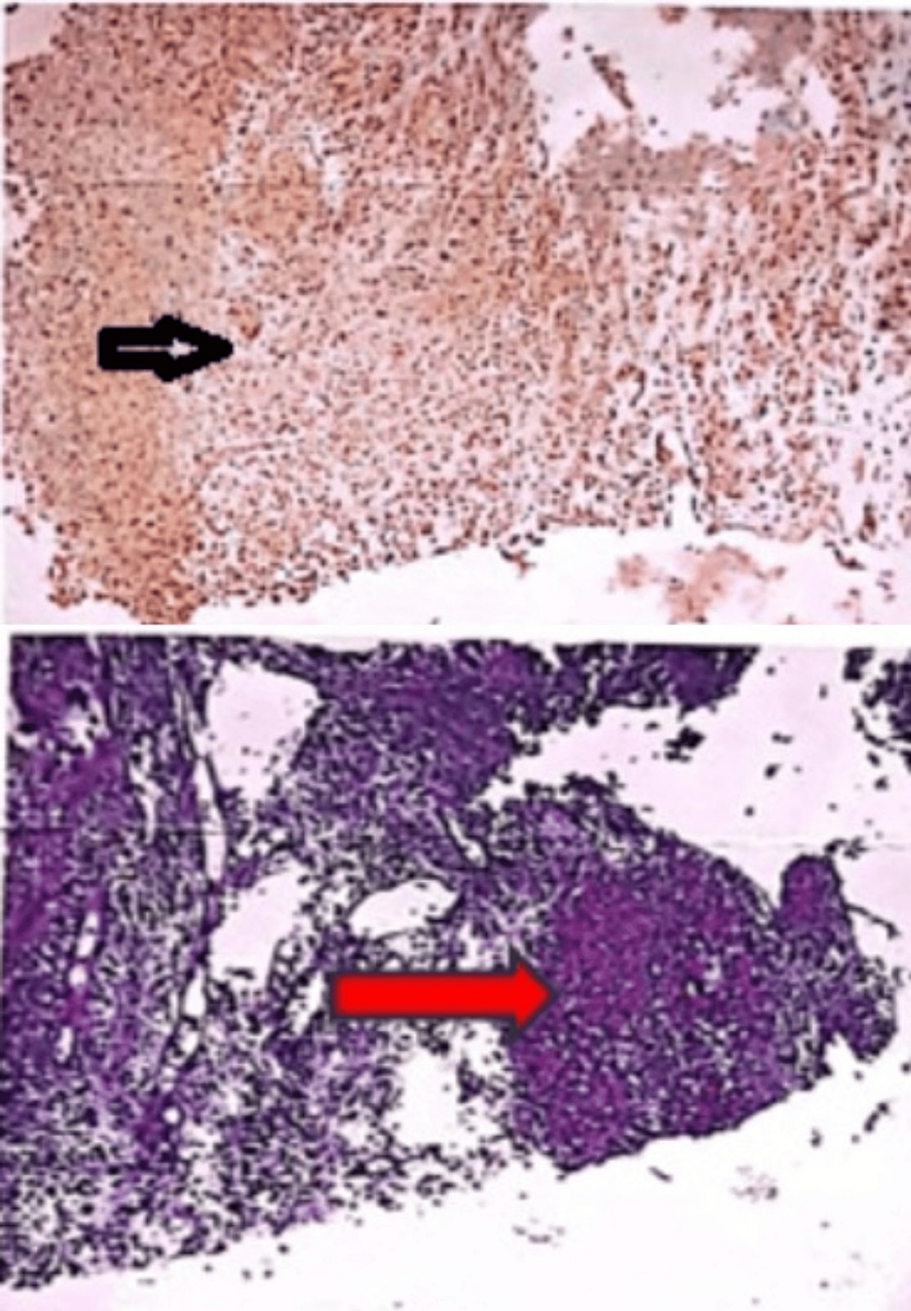
Liver tissue (core biopsy): granulomatous inflammation, histologically tuberculous Microscopic description: Section A: immunocytochemistry findings of Mycobacterial antigen; Section B: fibrocollagenous tissue. It presents areas of caseation surrounded by a focal collection of epitheloid cells, Langhans giant cells, and lymphocytes. No malignancy is seen.

A liver biopsy showed granulomatous inflammation consistent with tuberculous etiology, and immunohistochemistry confirmed the presence of *Mycobacterium tuberculosis* antigen with CBNAAT-confirmed *Mycobacterium tuberculosis* antigen (Figures 4A, 4B). Based on the clinical and histopathological findings with culture, she was diagnosed with an isolated tubercular liver abscess.

**Figure 5:**
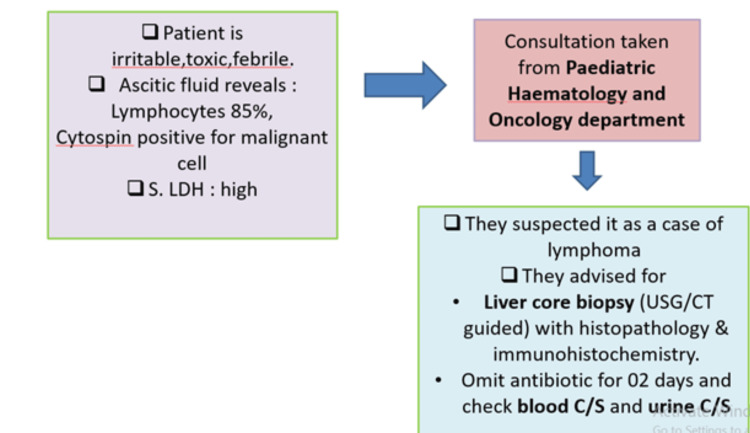
The treatment course for our patient on day six of hospitalization S. LDH: Serum lactate dehydrogenase; USG: Ultrasonogram and CT: Computed Tomography, Blood C/S: Blood culture, Urine C/s: Urine culture

In summary, a patient presented to the unit with a toxic appearance and fever, which is indicative of probable sepsis arising from an underlying condition. Prompt action was taken to conduct all necessary investigations, including an ascitic tap, which raised suspicion for malignancy due to high leukocytosis and LDH levels (a non-specific marker for tissue injury and infection). With the pediatric hemato-oncologist suspecting lymphoma as abnormal cells were positive for ascitic tap, a liver biopsy was planned, revealing tubercular pathogens through immunohistochemistry stains (Figure 5).

**Figure 6:**
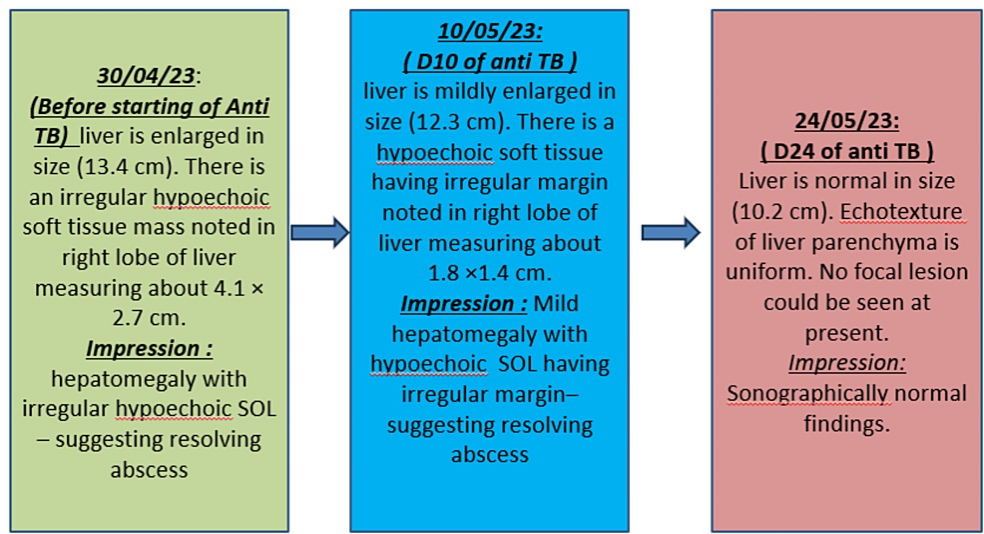
Serial ultrasonogram report summary with adequate treatment of tubercular abscess of the liver D10: Day 10; cm: Centimeter, D24: Day 24; SOL: Space occupying lesion

The initial two sets of blood cultures were negative. During the diagnostic process, antibiotics were temporarily discontinued for further assessment via blood and urine cultures. Following the diagnosis and the initiation of Rifampicin, Isoniazid, Ethambutol, and Pyrazinamide, medication toxicity was vigilantly monitored. The patient was followed up with blood work and a serial ultrasonogram (USG) (Figure 6).

**Figure 7:**
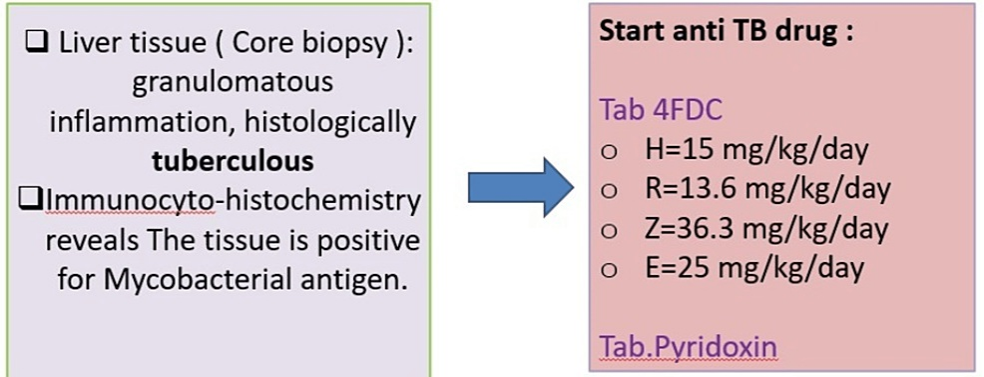
Treatment plan was initiated according to protocol for six months

The patient showed improvement after five days of anti-tubercular medications, and the treatment plan was outlined to adhere to the established protocol as tolerated (Figure 7).

**Figure 8:**
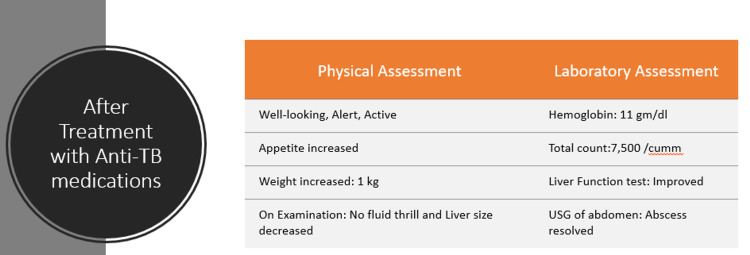
Summarized follow-up after 14 days with anti-tubercular treatment Anti-TB: Anti-Tuberculosis treatment; Kg: Kilogram; USG: Ultrasonogram

The patient was discharged on day 27 of hospitalization following an eleven-day course of antitubercular treatment. The prescribed anti-TB drugs are to be continued for a total duration of six months. Additionally, the patient is advised to take vitamins and minerals as part of the ongoing therapeutic plan. A follow-up assessment, including a complete blood count (CBC), serum glutamic-pyruvic transaminase (SGPT), C-reactive protein (CRP), and an ultrasound of the whole abdomen (USG of W/A), is scheduled after 14 days to monitor treatment progress and overall health status (Figure 8).

## Discussion

In a 2011 systematic review of 18 studies involving 1638 children with FUO, most studies required a minimum fever duration of two to three weeks for inclusion. The primary causes of FUO in this review were as follows [4]: Infections comprise 51% of cases, with 59% of these being bacterial infections. The specific bacterial infections varied by region; for instance, *Bartonellosis* and urinary tract infections were more prevalent in resource-rich countries, while resource-poor countries had a higher incidence of Brucellosis, tuberculosis, and typhoid fever.

Approximately 23% of cases remained undiagnosed or resolved before a diagnosis could be established. Rheumatologic diseases accounted for 9% of cases, with juvenile idiopathic arthritis and systemic lupus erythematosus (SLE) as the most frequently identified conditions in this category. Neoplastic disorders represented 6% of cases, with leukemia and lymphoma being the most common malignancies observed in children with FUO [4]. In our case report, the patient, previously treated at a different facility for unexplained fever, underwent imaging that raised suspicion of a liver abscess. A subsequent biopsy confirmed a diagnosis of tubercular liver abscess, and the initiation of treatment resulted in the resolution of the fever.

Pyogenic liver abscess typically manifests with fever, right upper quadrant pain and tenderness, and elevated liver enzymes. Most pyogenic liver abscesses are polymicrobial; mixed enteric facultative and anaerobic species are the most common pathogens. In a population-based analysis of pyogenic liver abscesses in children, *streptococcal* and *staphylococcal* species emerged as the predominant pathogens. The most effective method for differentiating amebic abscesses from pyogenic liver abscesses is through serological testing for *E. histolytica* [5]. Pyogenic liver abscesses frequently stem from portal vein pyemia, often associated with bowel leakage and peritonitis. Another significant pathway is direct extension from biliary infection, notably in cases of underlying biliary tract disease like gallstones or malignant obstruction. Additionally, liver abscesses can arise from hematogenous spread from the systemic circulation [5]. However, cases of isolated liver abscesses without an obvious source are rare in medical literature.

Diagnosing an isolated tubercular liver abscess can be challenging due to its nonspecific clinical presentation and the lack of specific serological markers. In a thorough assessment of 40 children with FUO referred to a pediatric tertiary clinic, despite occasional abnormal results in laboratory, radiological, and pathological studies, none led to a specific diagnosis [6]. Laboratory investigations, including a complete blood count, liver function tests, and inflammatory markers, may show nonspecific abnormalities. Imaging modalities, such as ultrasound and computed tomography (CT), play a crucial role in the diagnosis. The imaging features of an isolated tubercular liver abscess typically include solitary or multiple hypoechoic or hypodense lesions with central necrosis. The hypodense focal lesions observed on CT scans may be mistaken for amoebic or pyogenic liver abscesses or hepatomas. Therefore, a definitive diagnosis is established by confirming the presence of acid-fast bacteria (AFB) in pus, aspirate, or biopsy specimens. While *M. tuberculosis* culture is considered the gold standard for bacterial detection, it requires viable microorganisms and an extended incubation period of approximately 6-8 weeks.

Tuberculosis predominantly affects the lungs, lymph nodes, bones, and skin. However, extrapulmonary tuberculosis, including liver involvement, can occur in both adults and children, although it is a relatively infrequent occurrence, particularly among the pediatric population. Tuberculous liver abscesses are rare and typically present as multiple small abscesses (miliary tuberculosis). However, it should be contemplated as a possibility in individuals with a history of potential exposure when conventional pyogenic bacteria are not isolated from liver aspirate cultures [7,8]. Isolated tubercular liver abscess refers to the localized accumulation of pus within the liver parenchyma due to *Mycobacterium tuberculosis* infection without evidence of active tuberculosis elsewhere in the body, which is extremely rare, with a prevalence of 0.34% [9]. However, Bangladesh is still considered a TB-endemic region, and the atypical presentation of tuberculosis cases is visible in tertiary health care centers.

The management of isolated tubercular liver abscesses in children involves a multidisciplinary approach, including pediatricians, infectious disease specialists, and interventional radiologists. Antitubercular therapy, consisting of multiple drugs, such as isoniazid, rifampicin, pyrazinamide, and ethambutol, is the mainstay of treatment. Due to the infrequent occurrence of isolated tubercular liver abscesses in children and the complexities involved in diagnosing them, reporting such cases can enrich the medical literature and improve our comprehension of this ailment. It is crucial to include isolated tubercular liver abscess in the list of potential diagnoses for pediatric patients with unexplained fever, weight loss, and abdominal symptoms, especially in areas with a high prevalence of tuberculosis.

## Conclusions

This case highlights the diagnostic challenges of isolated tubercular liver abscess in children and the importance of considering this condition in pediatric patients presenting with unexplained fever, weight loss, and abdominal symptoms. Early recognition, appropriate diagnostic investigations, and the timely initiation of antitubercular therapy are crucial for successful management and improved patient outcomes.
